# Hypoxia in CNS Pathologies: Emerging Role of miRNA-Based Neurotherapeutics and Yoga Based Alternative Therapies

**DOI:** 10.3389/fnins.2017.00386

**Published:** 2017-07-11

**Authors:** Gillipsie Minhas, Deepali Mathur, Balakrishnan Ragavendrasamy, Neel K. Sharma, Viraaj Paanu, Akshay Anand

**Affiliations:** ^1^Neuroscience Research Lab, Department of Neurology, Post Graduate Institute of Medical Education and Research Chandigarh, India; ^2^Faculty of Biological Sciences, University of Valencia Valencia, Spain; ^3^Molecular Biosciences Laboratory, Swami Vivekananda Yoga Anusandhana Samsthana University Bengaluru, India; ^4^Armed Forces Radiobiology Research Institute Bethesda, MD, United States; ^5^Government Medical College and Hospital Chandigarh, India

**Keywords:** hypoxia, ischemia, microRNA, yoga, breathing exercise, neuroprotection

## Abstract

Cellular respiration is a vital process for the existence of life. Any condition that results in deprivation of oxygen (also termed as hypoxia) may eventually lead to deleterious effects on the functioning of tissues. Brain being the highest consumer of oxygen is prone to increased risk of hypoxia-induced neurological insults. This in turn has been associated with many diseases of central nervous system (CNS) such as stroke, Alzheimer's, encephalopathy etc. Although several studies have investigated the pathophysiological mechanisms underlying ischemic/hypoxic CNS diseases, the knowledge about protective therapeutic strategies to ameliorate the affected neuronal cells is meager. This has augmented the need to improve our understanding of the hypoxic and ischemic events occurring in the brain and identify novel and alternate treatment modalities for such insults. MicroRNA (miRNAs), small non-coding RNA molecules, have recently emerged as potential neuroprotective agents as well as targets, under hypoxic conditions. These 18–22 nucleotide long RNA molecules are profusely present in brain and other organs and function as gene regulators by cleaving and silencing the gene expression. In brain, these are known to be involved in neuronal differentiation and plasticity. Therefore, targeting miRNA expression represents a novel therapeutic approach to intercede against hypoxic and ischemic brain injury. In the first part of this review, we will discuss the neurophysiological changes caused as a result of hypoxia, followed by the contribution of hypoxia in the neurodegenerative diseases. Secondly, we will provide recent updates and insights into the roles of miRNA in the regulation of genes in oxygen and glucose deprived brain in association with circadian rhythms and how these can be targeted as neuroprotective agents for CNS injuries. Finally, we will emphasize on alternate breathing or yogic interventions to overcome the hypoxia associated anomalies that could ultimately lead to improvement in cerebral perfusion.

## Introduction

Mammalian brain lacks fuel reservoirs and hence needs a constant supply of glucose for ATP generation, cell survival, and production of neurotransmitters. Although it constitutes only 2% of body weight, it consumes nearly a quarter of total body glucose (~5.6 mg glucose per 100 g human brain tissue per minute) (Erbslöh et al., 1958). Under physiological aerobic conditions, glucose is completely metabolized into carbon dioxide and water resulting in the formation of ATP. Any hindrance in the oxygen supply to the brain may cause catastrophic effects to brain cells. In this review we will first discuss the pathophysiological effects caused due to hypoxia, which has been associated with different neurodegenerative diseases and consecutively, we will discuss miRNA based therapeutic approach to target the hypoxia-associated CNS injuries along with alternate therapies for management of hypoxia.

## Pathophysiological responses to hypoxia

Oxygen is an essential element for cell survival. It acts as a final electron acceptor in oxidative phosphorylation, which ultimately leads to the production of energy in the form of ATP. However, under hypoxia, a cell undergoes prolonged energy deprivation that results in irreversible cellular dysfunction and ultimately leads to cellular demise (Gutierrez, 1991; López-Barneo et al., 2001; Hardie, 2003). Hypoxia may occur in both basal conditions as well as diseased conditions (Brahimi-Horn et al., 2007; Brahimi-Horn and Pouysségur, 2007; Sluimer et al., 2008; Li Z. et al., 2009).

In response to hypoxia, a cell triggers a plethora of reactions, which include disturbance in ion channel homeostasis, energy failure, free radical production, etc. It has been estimated that 60% of ATP is consumed by ATPases, such as the Na^+^/K^+^ ATPase, and Ca^2+^ATPase under normal conditions (Zauner et al., 2002). During hypoxia, there is a reduction in the intracellular ATP/ADP ratios, which disrupts the ion concentration gradients by causing an increased efflux of K^+^ ions and influx of Na^+^ and Ca^2+^ ions. Eventually, membrane depolarization takes place and the accumulated Ca^2+^ ions activate the proteases, which leads to membrane damage, disturbance in mitochondrial metabolism, and generation of reactive oxygen species (ROS) (Boutilier, 2001). Under prolonged exposure to hypoxia, hypoxia-inducible factor (HIF) is upregulated, which is a well-studied transcription factor and serves as a potential endogenous marker for hypoxia. HIF-1 transcriptional complex, a ubiquitously expressed protein made up of two subunits namely HIF-1α and HIF-1β which binds to DNA at hypoxia response elements (HRE) of target genes (Semenza et al., 1994; Semenza, 1999; Kemp and Peers, 2007). More than 200 target genes have been known to be regulated by the HIF complex, which further “turns on” factors involved in erythropoiesis, pro-inflammatory gene expression, energy metabolism or apoptosis, angiogenesis, and cellular survival and proliferation (Kaur et al., 2005; Lundgren et al., 2007; Loor and Schumacker, 2008). Age is a major risk factor for hypoxia and associated clinical outcomes. The vulnerability to hypoxia-induced diseases increases many-fold with age. It has become more evident that with age the oxygen pressure falls which along with a lower cerebral perfusion results in microglial activation and neuroinflammation (Buga et al., 2013; Popa-Wagner et al., 2014; Sandu et al., 2015). Age-related changes in HIF have also been established that have been associated with increased susceptibility toward stroke (Katschinski, 2006). Frenkel-Denkberg et al. observed a difference in DNA-binding efficiency of HIF when compared between young and old mice that were exposed to hypoxia (Frenkel-Denkberg et al., 1999). Different studies have demonstrated that the aging theories of telomere shortening and free-radical generation, both are dependent on the availability of oxygen (Nishi et al., 2004). It has been seen that by conducting aerobic exercise processes including yoga, there is an increase in the expression of brain-derived neurotrophic factor (BDNF) in the body. BDNF is an important mediator of neurogenesis as well as neuronal plasticity. It promotes growth in various areas of the brain such as the hippocampus, the dorsal root ganglions as well as cortical neurons. These levels decrease with age, however an increase in all age groups which performed aerobic exercises, in this case yoga, was observed (Pal et al., 2014). Aerobic exercises increasing blood oxygen levels and thus promoting cerebral perfusion have shown to have a direct impact in the reduction of Sympathetic Adrenal Medulla axis (SAM). Activation of SAM causes an increased release of catecholamines from the adrenal medulla. In acute scenarios, these catecholamines are of great importance. However, if chronically raised they can lead to increased vascular resistance, which in turn can lead to decreased perfusion of organs including cerebral perfusion (Arora and Bhattacharjee, 2008).

Literature indicates that cell differentiation is promoted upon hypoxia in different cell types. It has been demonstrated that molecules playing a critical role in cell differentiation such as Notch, MYC, and Oct-4 in conjunction with hypoxia and HIFs are associated with each other (Simon and Keith, 2008). However, the molecular mechanism and pathways by which hypoxia induces cell differentiation is poorly understood (Gustafsson et al., 2005; Samanta et al., 2017). It is also reported that hypoxia directly regulates the Notch signaling pathway activity in the cell differentiation process (Gustafsson et al., 2005). Additional molecules which are directly influenced by HIF-2α include OCT4 and MYC transcription factors (Dang et al., 2008; Simon and Keith, 2008). By modifying cell growth, cells respond to oxygen deprivation metabolism by activation of hypoxia induced genes. In various cell types the role of HIF1α and hypoxia has been investigated in cellular proliferation. Under hypoxic conditions Notch signaling is also increased so as to maintain progenitor/stem cell state, which is dependent on HIF-1α accumulation (Pear and Simon, 2005). Hypoxia leads to increased Notch1 signaling which further leads to differential expression of regulatory cell cycle proteins and increased proliferation of cells. However, how HIF-1α leads to the activation of these transcription factors are poorly understood and needs further investigations.

Moreover, hypoxia has also been shown to have an impact on circadian rhythms for temperature and oxygen levels. A study by Mortala et al. has demonstrated disruption in temperature rhythms in rats exposed to different levels of oxygen under 12 h light-dark (LD) cycle vs. those under constant light regimen, suggesting a connection between hypoxia and circadian rhythms (Mortola and Seifert, 2000). Likewise, it has also been noted in conditions increasing oxygen transport to the brain such as in aerobic exercise and controlled breathing exercises that there is an increase in alpha wave activity as well as a decreased galvanic skin response. Both of these parameters facilitate a more steady and consistent circadian rhythm, which in turn maintain a more steady oscillation of temperature and oxygen levels (Kumar and Joshi, 2009).

A number of studies have also demonstrated that both pre- and post-hypoxic conditioning could offer protective effects to the tissue and hence may function as a novel therapeutic strategy. Hypoxic/ischemic preconditioning is performed by exposing the tissue to hypoxia/ischemia below dangerous levels for a considerable number of times so that tissue becomes resistant to subsequent insult. This process protects the tissue from further injury. Similarly, post-hypoxic conditioning shields the tissue from damage as it develops tolerance against hypoxia through repeated inductions but is applied after the injury has occurred. The therapeutic effects and mechanisms of hypoxic preconditioning were reported in the 1960s much earlier than cerebral ischemic preconditioning was studied but were not emphasized to that extent as the latter one (Lu, 1963; Dahl and Balfour, 1964). Later, Schurr et al. studied hypoxic preconditioning and brain tolerance in rat hippocampal slices (Schurr et al., 1986). Numerous other *in vitro* models of hypoxic preconditioning have been studied such as primary neuronal cultures, hippocampal slices, olfactory cortex, and transient ischemic attacks in humans showing tolerance effects against the pathological state (Kirino et al., 1996; Perez-Pinzon et al., 1996; Arthur et al., 2004; Steiger and Hanggi, 2007; Obrenovitch, 2008; Shpargel et al., 2008; Bickler and Fahlman, 2009; Gidday et al., 2013).

Hypobaric hypoxia is another means of inducing hypoxic preconditioning and its neuroprotective potential and other beneficial health related effects have already been demonstrated (Meerson, 1984; Meerson et al., 1996; Gong et al., 2012; Millet et al., 2013; Zhen et al., 2014). Rybnikova et al. employed this technique (Samoilov et al., 2001; Rybnikova et al., 2005, 2006) to investigate its mechanisms and therapeutic potential. Recently, same group of investigators used this model in rats and found that both early and delayed applications of the post-conditioning augmented recovery and mitigated neuronal injury caused as a result of hypoxia (Rybnikova et al., 2012). Similarly, Gamdzyk et al. found neuroprotective potential of this technique in the rat model (Gamdzyk et al., 2014). Ischemic post-conditioning is also proven to protect heart and brain tissues (Zhao et al., 2003; Zhao, 2007). All these data suggest the therapeutic potential of hypoxic pre- and post-conditioning and need further investigation. Consequently, the complex nature of pathophysiology associated with hypoxia provides numerous check-points that could be targeted as therapeutic targets.

## Hypoxia/ischemia in CNS pathologies

The reduced blood supply also deprives the tissue from oxygen and nutrients leading to tissue damage and cell death in a prolonged hypoxic state. Under normal scenario, the body has its own mechanisms to sense oxygen deprivation and to overcome from this. However, permanent damage and pathological outcome may arise in conditions where the tissue under hypoxia is unable to reverse the deficiency. Brain, as it is well-known, has a very high metabolic demand and is completely dependent on oxygen supply for glucose metabolism. Any small disruption in oxygen supply, even for a few seconds, can cause sudden and irreversible damage in the functioning of the brain. Hypoxia in CNS tissues can initiate a series of pathophysiological events that can exert their own impact.

Furthermore, hypoxic injury has been associated with many clinical CNS anomalies including stroke, encephalopathy, ischemic retinopathies, and Alzheimer's (Peers et al., 2007; Zhang and Le, 2010). However, previous studies related to hypoxia and Alzheimer's disease (AD) are meager. With recent investigations it has come into light that the pathology of AD shares common links with hypoxia/ischemia (Peers et al., 2007; Lanteaume et al., 2016; Liu et al., 2016). Studies have demonstrated that hypoxia induces up-regulation of beta-secretase 1 (BACE1) gene expression both *in vitro* and *in vivo*, thereby contributes to the pathology of Alzheimer's disease (AD) (Sun et al., 2006). A recent finding also demonstrates that hypoxia induces epigenetic alteration in AD and suggests that it can acerbate AD progression through demethylation of genes encoding γ-secretase enzyme (Liu et al., 2016). This study revealed that hypoxia exaggerated the neurological outcome and memory dysfunction in AD mice. Patients suffering from stroke or cardiovascular disease have been shown to be more susceptible to cognitive impairment and dementia, such as Alzheimer's (de La Torre, 2008). Zhang et al. demonstrated recently that miR-124 regulates the expression of BACE1 in the hippocampus under chronic cerebral hypoperfusion (Zhang et al., 2017). In case of cerebral ischemia, the susceptibility toward AD was shown to increase (Jendroska et al., 1995; Altieri et al., 2004). Hypoxia can also induce formation of amyloid β plaques on prolonged exposure. Studies conducted in different cell lines exposed to hypoxic conditions demonstrate increase in production of amyloid regardless of the cell line being tested (Wang et al., 2006; Li L. et al., 2009; Muche et al., 2015). The mechanism behind the pathogenesis of AD being linked with hypoxia/ischemia is still unknown. Nonetheless, it has been revealed that the pathways of calcium homeostasis and calcium channel signaling are the connecting link between the AD prognosis and hypoxia (Green and Peers, 2001; Bai et al., 2015). Apart from increasing the production of amyloid β plaques, it has been shown that hypoxia also leads to other pathological hallmarks associated with AD, such as tau phosphorylation, decreasing the degradation, and clearance of plaques (Burkhart et al., 1998; Fang et al., 2010; Zhang and Le, 2010; Zhang et al., 2014).

Recently, Ashok et al. demonstrated that hypoxia-inducible factors may be neuroprotective in Alzheimer's disease (Ashok et al., 2017). Furthermore, hypoxia and ischemia activates processing of amyloid precursor protein and plays a crucial role in the pathogenesis of AD (Salminen et al., 2017). Several investigations using cellular models and *in vivo* studies have revealed that expression and activity of neprilysin (NEP), which is an Aβ-degrading enzyme, declines with age and also under hypoxic/ischemic conditions. This may result in accumulation of Aβ plaques and eventually development of AD pathology (Nalivaevaa et al., 2004; Caccamo et al., 2005; Wang et al., 2005; Fisk et al., 2007; Nalivaeva et al., 2011, 2012; Zhuravin et al., 2011). Furthermore, the levels of caspases such as caspase 3, 8, and 9 are markedly increased in human neuroblastoma NB7 cells, which can cleave APP intracellular domain (Kerridge et al., 2015). These findings indicate that NEP levels in hypoxic/ischaemic brain might be regulated by stimulation of caspases, which may result in loss of Aβ clearance and related to development of AD.

In case of encephalopathy, an obstruction in cerebral blood flow leads to oxygen/glucose shortage in an infant's brain and eventually primary energy failure (Shalak and Perlman, 2004). Simultaneously there is an increase in the production of lactate due to a switch from aerobic to anaerobic metabolism (Hanrahan et al., 1996). A cascade of reactions antecede this event such as disturbance in sodium/potassium (Na^+^/K^+^) pumps, Ca^2+^ influx, membrane depolarization, glutamate release, and subsequently cellular death (Johnston et al., 2009). Both apoptotic and necrotic cell death have been visualized in primary and secondary energy failure during neuronal injury with the later most prominently seen with primary energy failure (Cotten and Shankaran, 2010). Moreover, during the injury immune infiltrates penetrate the blood-brain barrier (BBB) and enhances neuronal injury but the mechanism remains elusive (Ferriero, 2004). Palmer et al. documented that neutrophils invade the BBB in the initial phase and causes brain edema (Palmer et al., 2004). Therefore, involvement of hypoxia in neuronal degeneration insists the need to explore the therapeutic targets that will target common pathological hallmarks and consequently will help in ameliorating these hypoxia-induced disease conditions.

## Development of novel therapeutic intervention: miRNA in neuroprotection

Hypoxia associated CNS anomalies, such as stroke, Alzheimer's, ischemic retinopathy, and encephalopathy are all linked with some common pathophysiological attributes that could be targeted for therapeutics. With discovery of miRNA-mediated regulation of gene expression, many studies have investigated the miRNA as interventions for human diseases, with few studies now being conducted at preclinical and clinical levels (Christopher et al., 2016).

Over the last 10 years, miRNA have been identified as the 18–25 nucleotides long non-coding RNA molecules responsible behind the translation regulation. These have been observed in organisms from Drosophila to humans (Bartel, 2004). In human genome miRNA have been shown to control various physiological and pathophysiological processes. A functional miRNA is formed in a two-step process, from a primary miRNA or pre-miRNA, which is transcribed through RNA polymerase II. It is then further processed into a hairpin structure (70–100 nucleotide long) by ribonuclease III Drosha that is complexed with RNA binding protein Pasha. Ultimately this hairpin structure is transported to cytoplasm, where it is acted upon by Dicer to form the mature 18–25 nucleotide long miRNA (Lee et al., 2003, 2004). How these miRNAs work to regulate gene expression is a complex process with still many aspects unknown. Mostly these miRNA exert their action through the formation of RNA-induced silencing complex (RISC). Typically the miRNA exert their effect by binding through RISC to the target genes at their 3′ UTR sites and regulating the levels of proteins, either by mRNA degradation, translation repression, or decapping of mRNA (Macfarlane and Murphy, 2010). These negatively regulate the post-transcriptional expression of their target genes either through blocking the translation of concerned mRNA or by degrading them (Ivan et al., 2008). Another aspect essential for the miRNA to function is the sequence complementarity between the miRNA and the mRNA, which decides the fate and mechanism for mRNA regulation. Moreover, a single miRNA can control multiple targets and vice versa, multiple miRNA molecules can collectively regulate a single target mRNA (Bartel, 2004).

miRNAs play a crucial part in development and functioning of brain (Meza-Sosa et al., 2012; Davis et al., 2015). Several brain-specific miRNA have been identified in mouse as well as humans, which include miR9, miR124a, miR124b, miR135, miR153, and others (Sempere et al., 2004). miRNAs serve as vital regulators in the central nervous system (CNS) and are implicated in various neurological diseases (Cao et al., 2016). Indeed, atypical levels of miRNA have been observed in many neurological disorders including those that are hypoxia induced. A few these miRNA that have been identified to play an important role in ischemic injury, include miRNA 15, miRNA 21, miRNA 29, miRNA 124, miRNA 145, miRNA 181, miRNA 200 family, miRNA 497, and others (Xiao et al., 2015). miRNA profiling studies in different animal models of ischemia along with human patients have been conducted, which have revealed patterns of miRNA expression at different time-points post-injury (Jeyaseelan et al., 2008; Tan K. S. et al., 2009). miRNA 210 is one that has shown conservation throughout the evolution. It is expressed in hypoxic tissues and is activated by HIF1α, a hypoxia inducible transcription factor (Chan and Loscalzo, 2010). The expression has also been revealed in middle cerebral artery occlusion (MCAO) model in rat brain as well as blood (Zeng et al., 2011).

Since the role of miRNA has been implicated in neuronal development as well as disease pathogenesis, it becomes necessary to explore the miRNA-associated regulation as a potential to develop novel clinical biomarkers and therapeutics. Recent studies have identified miRNA derivatives, anti-miRNA oligonucleotides and locked nucleic acids, as therapeutic targets to be tested in clinical scenario (Weiler et al., 2006; Love et al., 2008; Nampoothiri et al., 2016). Qiu et al. established the neuroprotective role of miRNA 210 in hypoxia-ischemia injury, where the carotid artery in rats was permanently ligated at post-natal day 7, followed by exposure to hypoxia for 2 h. miRNA 210 is known to decrease the apoptosis of neuronal cells along with inhibition of caspases (Qiu et al., 2013). miRNA such as these can be targeted as novel therapeutics for ischemic injury. Hu et al. tested the delivery of miRNA 210 through a non-viral vector in ischemic heart injury and demonstrated a decrease in apoptosis (Hu et al., 2010). In different studies it has also been shown that it easily crosses the BBB. Moreover, investigations have also revealed miRNAs that have been implicated to confer neuroprotection in Alzheimer's disease (Liu et al., 2012; Yang et al., 2015). The development of neurodegenerative diseases is associated with the differential expression of miRNAs. Earlier investigations have revealed that ischemic stroke can trigger variations in the miRNA expression (Kocerha et al., 2009; Tan K. S. et al., 2009). A study investigating changes in miRNA profiling showed that 138 miRNAs were upregulated and 19 miRNAs were downregulated in stroke patients (Cao et al., 2012). In addition, miRNAs including hsa-let-7f, miR-126, −1259, −142-3p, -15b, -186, -519e, and -768-5p were found to be downregulated in different subtypes of stroke. Likewise, miRNAs including hsa-let-7e, miR-1184, −1246, −1261, −1275, −1285, −1290, −181a, −25^*^, −513a-5p, −550, −602, −665, −891a, −933, −939, and −923 showed upregulated expression in the stroke patients (Tan et al., 2013).

An interesting study showed that miRNA expression is sex-specific in ischemic stroke patients (Siegel et al., 2011). A significantly elevated expression of miR-23a was found in females whereas reduced expression was observed in males' post-ischemic event. Furthermore, upregulated expression of miR-223 confers neuroprotection in stroke patients by reducing the expression of the glutamate receptor subunits (Harraz et al., 2012). Alternatively, its decreased expression may aggravate neuronal cell death by elevating glutamate receptor subunits levels thereby indicating that it can prevent neuronal injury by regulating the expression profiles of glutamate receptor subunits. In addition, reduced expression of miR-145 may also prevent neuronal cell death by increasing superoxide dismutase-2 levels (Dharap et al., 2009). Let7f and miR1 was shown to subdue neuroprotection by controlling insulin-like growth factor 1, which protects neurons endogenously (Selvamani et al., 2012). Conversely, treatment with anti-miR1 and anti-Let7f are known to significantly reduce the infarct volume and protect neurons from cell death post-stroke. The findings of Buller et al. (2010) showed that expression of miR-21 is enhanced by three-folds following stroke *in vivo*. miR-21 overexpression inhibits apoptosis and mediates neuroprotection *in vitro*. A study reported that miR-181 expression changes in stroke, and reduced expression of miR-181 confers neuroprotection by manipulating glucose-regulated protein-78 (GRP78) (Ouyang et al., 2012). In addition, miR-124a, miR-210, miR-125b, and anti-inflammatory (miR-26a, miR-34a, miR-145, and let-7b) miRNA show altered expression profiles in stroke patients (Liu et al., 2011; Rink and Khanna, 2011; Zeng et al., 2011). In Alzheimer's disease, expression profiles of several miRNAs have been found to be altered in both the human AD tissue and diseased model (Cogswell et al., 2008; Hebert et al., 2008; Lukiw et al., 2008; Patel et al., 2008; Wang et al., 2008; Croce et al., 2013) suggesting that dysregulated miRNAs are associated with AD pathogenesis. Table 1 summarizes different miRNA shown to regulate hypoxia/ischemia and thus, their neuroprotective potential in CNS injuries.

**Table 1:** Different miRNA known to regulate hypoxia/ischemia associated genes and how these can play a neuroprotective role in CNS pathologies.

**miRNA**	**Associated target/Role in CNS injury**	**Neuroprotective role**	**References**
miR 21	Blocks Fas ligand; increased after ischemia	Increases neuronal survival post ischemia	Buller et al., 2010
miR 30a, miR 383, miR 320a	Aquaporin proteins; downregulated in ischemia	Overexpression can modulate cerebral edema	Jeyaseelan et al., 2008
miR-106	Regulates the transporter ABCA1 involved in ApoE production	In AD patients downregulated in the temporal cortex	Kim et al., 2012
miR-107	Up regulation of BACE1	Downregulated in temporal cortex of AD patients which could impact upon Aβ production	Goodall et al., 2013
miR-124	Regulates the Expression of BACE1	In the hippocampus under chronic cerebral hypoperfusion	Long et al., 2014
miR-125b	Cell cycle regulator	Glial cell and astroglial proliferation	Pogue et al., 2010
miR 126, miR 130a, miR 296, miR 424	Promote angiogenesis	Modulation can increase angiogenesis after ischemia and maintain vascular integrity	Würdinger et al., 2008; Ghosh et al., 2010; Caporali and Emanueli, 2012
miR-134	Heat-shock proteins; increased in ischemia	Downregulation can decrease apoptosis and cellular damage, improve neurological outcomes	Chi et al., 2014
miR-142 -3p	Promotes the IL-1β-dependent glutamate dysfunction	Upregulated in the CSF of MS patients and in experimentl autoimmune encephalomyelitis cerebellum	Mandolesi et al., 2017
miR-145	Targets SOD2; Upregulated after ischemia	Antagonists can increase SOD2 expression and decrease ROS	Dharap et al., 2009
miR-146a	Complement activation repressor	Altered innate immune response and neuroinflammation	Alexandrov et al., 2011
miR-146a	Transmembrane protein; regulator of βAPP cleavage	Aberrant βAPP processing and amyloidogenesis	Yanez-Mo et al., 2011
miR 181	Glucose-regulated protein 78 (GRP78); increased in ischemic injury	Reduction can increase neuronal survival	Ouyang et al., 2012
miR 155	Regulates inflammation	Regulates CD4^+^ and CD8^+^ T cell accumulation, NK cell maturation and expansion, T cell cytokine production, CD8^+^ T cell-mediated cytotoxicity, astrogliosis, macrophage polarization, expression of receptors necessary for viral entry, and expression of viral proteins	Dickey et al., 2017
miR 210	Decreased in ischemic stroke	Overexpression induces angiogenesis	Fasanaro et al., 2008; Zeng et al., 2011; Lou et al., 2012; Ma et al., 2016
miR-339-5p	Regulates BACE1 expression and is most likely dysregulated in AD patients	Triggers the amyloidogenic pathway	Long et al., 2014
miR 497	Anti-apoptotic genes—Bcl2; upregulated in ischemia	Inhibition increases neurological function and decreases infarcts	Yin et al., 2010

Moreover, the function of miRNA as a master regulator of gene expression has made it a potential for clinical translation. For instance, the first clinical trial was with miR 122 antagonists against Hepatitis C virus is under Phase II trial (www.clinicaltrials.gov/NCT01200420) (Gebert et al., 2013). Another clinical trial registered at ClinicalTrials.gov is for miR 34 in hepatic cancers (www.clinicaltrials.gov/NCT01829971). In Alzheimer's disease, a clinical trial investigating correlation between miR 107 and BACE1 levels has been initiated (www.clinicaltrials.gov/NCT01819545). Apart from these, anti-miRNA drugs have also been developed for cardiac as well as muscular disorders (Hydbring and Badalian-Very, 2013). All these clinical trials and drugs have paved way for much more clinical translation to come in near future.

Nonetheless there are some issues that need more investigations such as route of delivery, effects that can be off-target and safety and can be resolved before translation to clinics.

### miRNA in inflammation and angiogenesis

Hypoxia/ischemia is a complex phenomenon, with multiple events that play a role in the pathogenesis including energy failure, ROS generation, inflammation, and angiogenesis. It has been seen that conditions increasing cerebral perfusion as in aerobic exercises, such as yoga, practiced over a long period of time actually reduce serum IL-6 levels, a pro-inflammatory cytokine found in the body. There was also an increased presence found of C-reactive protein, another inflammatory species in the body, in those who did not perform these aerobic exercises (Kiecolt-Glaser et al., 2010). The two pathways that can be mainly targeted through regulation of miRNA includes angiogenesis and inflammation (Ouyang et al., 2015). Bonauer et al. investigated the role of miR 92a in angiogenesis *in vitro* in endothelial cells as well as *in vivo* using a mouse model of hind-limb ischemia and revealed that overexpressing the miR 92a under ischemic conditions inhibits angiogenesis, therefore, administration of an inhibitor could enhance the blood vessel growth and assist in recovery from ischemia (Bonauer et al., 2009). miRNA-210 is another one that is known to induce angiogenesis in cerebral ischemia. Lou et al. revealed increased expression of miRNA-210 in endothelial cells in a rat model of cerebral ischemia, which was shown to be through Notch 1 signaling, thus, indicating angiogenesis. In another study, overexpressing miR-210 lead to angiogenesis as well as neurogenesis in a mouse brain. miR-210 expression in neuronal cells demonstrated their role in proliferation of endothelial cells and formation of new vascular microvessels (Lou et al., 2012; Zeng et al., 2014). miRNAs are overexpressed in endothelial cells and they have been demonstrated to modulate angiogenesis and endothelial cell function. Angiogenesis is a process in which new blood vessels emanate from preexisting vessels. It has been reported by several studies that Dicer enzyme, if inactivated may result in abnormal endothelial cell function and blood vessel formation. Dicer is an enzyme involved in miRNA biogenesis and functions in regulation of vascular development (Yang et al., 2005; Kuehbacher et al., 2007; Suarez et al., 2007, 2008). Yang et al. showed that mice hypomorphic for Dicer die after few days and possess abnormal blood vessel formation suggesting that Dicer is required for normal mouse development (Yang et al., 2005). Several other groups observed profound consequences resulted by knockdown of Dicer, and concluded that molecule is necessary for normal mouse development (Bernstein et al., 2003). Giraldez et al. found that there were significant abnormalities in gastrulation phase, brain development, and blood circulation in zebrafish offspring model which was deprived of both maternal and zygotic Dicer (Giraldez et al., 2005).

It has been seen that angiogenic regulators such as angiopoietin-2 receptor, Tie-1, VEGF, and its receptors VEGFR1 and VEGFR2, are differentially expressed and linked with abnormal Dicer ex1/2 embryos and yolk sacs (Yang et al., 2005; Suarez and Sessa, 2009). Several other *in vitro* studies have determined the significant role of miRNAs in endothelial cells (Otsuka et al., 2007, 2008). Knockdown of Dicer in human endothelial cells lead to the formation of defective capillary-like structures and stunted cell growth (Kuehbacher et al., 2007; Suarez et al., 2007, 2008). In addition, expression of Tie-2/TEK, VEGFR2, endothelial nitric oxide synthase, IL-8, and angiopoietin-like 4 regulator proteins is found to be altered in Dicer silenced endothelial cells (Suarez et al., 2007). Interetingly, the expression of thrombospondin-1 remained elevated in these cells (Kuehbacher et al., 2007; Suarez et al., 2008).

Numerous *in vivo* studies demonstrated the crucial role of Dicer in postnatal angiogenesis. Findings of Kuehbacher et al. (2007) showed sprout formation was abridged when nude mice was subcutaneously injected for Dicer silencing in HUVECs (suspended in a Matrigel plug). Similarly, Suarez et al. (2008) generated two Dicer knockout mouse cell lines, which were endothelial specific (Suarez et al., 2008). These findings suggest that the role of miRNA in modulation of distinct aspects of angiogenesis might prove to be worthwhile in many human pathological diseases, especially those involving the vasculature.

Inflammation is an important impact as well as a cause in ischemia-reperfusion injury. Eisenhardt et al. in their study investigated the role of miRNA-155 in a mouse model of myocardial ischemia-reperfusion and revealed its involvement in exacerbation of inflammation as well as ROS generation post-injury (Eisenhardt et al., 2015). miRNAs play a crucial role in regulating both innate and adaptive immunity (Lu et al., 2009; Dalal and Kwon, 2010). Innate immunity is the first line of defense and protects the organism from invasion of foreign pathogens. On the contrary, adaptive immune system is highly specific to a particular antigen and elicits B and T lymphocytes to combat microbes. The former immune response neutralizes the effect of pathogens through the pathogen associated molecular patterns (PAMPs), recognition process by the toll-like receptors, or TLRs (Takeda and Akira, 2015). TLRs are transmembrane proteins expressed by cells participating in innate immune system, which recognize invading microbes and induce downstream signaling pathways that triggers immune responses to kill foreign pathogens.

It is well-documented that miRNAs are implicated in regulating inflammation and immune responses. A number of miRNAs have been induced by lipopolysaccharide (LPS) mediated TLR signaling such as miR-146a, miR-155, and miR-132 (Taganov et al., 2006). Studies have shown that cytokines like interleukin-1β, LPS, and tumor necrosis factor (TNF-α) stimulates the expression of miR-146a. Consecutively, miR-146a, suppresses the expression of IL-1 receptor associated kinase and TNF receptor-associated factor-6, components of the TLR4 signaling pathway (Taganov et al., 2006). Hence miR-146a negatively controls TLR signaling pathway. Similarly, interferon-β, and LPS induces the expression of miR-155 in murine macrophages (O'connell et al., 2007; Tili et al., 2007). Once miR-155 are activated, cytokines such as interleukin-6 (IL-6) and tumor necrosis factor-α (TNF-α) are triggered (Tili et al., 2007). miR-155 regulates suppressor of cytokine signaling (SOCS)-1 demonstrating its involvement in regulation of innate immune system (Rodriguez et al., 2007; Tili et al., 2007; Lu et al., 2009). Ceppi et al. showed that silencing of miR-155 in dendritic cells derived from human myeloid cells significantly elevated protein levels of the pro-inflammatory cytokine interleukin1 (IL-1) (Ceppi et al., 2009). In addition, miR-155 knockdown in these antigen-presenting cells directly suppressed pro-inflammatory TAK1-binding protein 2 levels, indicating its anti-inflammatory property (Ceppi et al., 2009). On the contrary, several studies have reported that miR-155 can augment inflammation. Overproduction of miR-155 in mouse bone marrow results in a myeloproliferative phenotype (O'connell et al., 2007).

While the expression of both miR-146 and miR-155 is elevated in macrophages in response to LPS stimulation, the expression of miR-125b is decreased. Tili and colleagues showed that reduced expression of miR-125b results in increased expression of TNF-α and eventually augments inflammation (Tili et al., 2007). Several other miRNAs such as miR-9, miR-21, and miR-147 are induced by TLR signaling and are expressed considerably and hence regulate immune responses in response to microbial invasion (Bazzoni et al., 2009; Liu et al., 2009; Sheedy et al., 2010).

Similarly, miRNAs are implicated in adaptive immune system by regulating the development of both T cell and B cell lymphocytes. Numerous studies have shown the involvement of miRNAs in controlling signaling networks in T cells (Monticelli et al., 2005; Wu et al., 2007; Merkerova et al., 2008). Wu et al. demonstrated that some overexpressed miRNAs are dynamically regulated during antigen-specific T-cell differentiation when miRNA profiling was performed in naive, effector and memory CD8+ T cells (Wu et al., 2007). Other studies showed that there were abnormally developed and fewer mature T-cells in Dicer deficient mice as compared to normal mice (Cobb et al., 2005; Muljo et al., 2005).

Reports have revealed the involvement of both miR-17 and miR-92 in T cell development (Xiao et al., 2008). Lately, it has been reported that miRNAs are also involved in the differentiation of T cells into distinct effector T helper cells. Du et al. reported that miR-326 regulates differentiation of TH17 cells both *in vitro* and *in vivo* (Du et al., 2009). Other miRNAs such as miR-181a can facilitate the intensity of T cell receptor signaling (Li et al., 2007) whereas miR-155 is implicated in regulatory T (Treg) cell formation and function (Zheng et al., 2007; Chong et al., 2008; Liston et al., 2008; Zhou et al., 2008). Furthermore, role of several miRNAs such as miR-150, miR-155, and miR-34a has also been described in humoral immunity involving B-lymphocytes (Chen et al., 2004; Monticelli et al., 2005; Vigorito et al., 2007; Xiao et al., 2007; Zhou et al., 2007; Basso et al., 2009; Tan L. P. et al., 2009; Xiao and Rajewsky, 2009; Rao et al., 2010).

Studies have indicated toward miRs that could regulate the immunological processes as well as nervous system, which have been termed as NeurimmiRs. NeurimmiRs such as miR 132 and miR 124 have shown to play a role in cross-talk between the two, at both local and peripheral levels. Furthermore, investigating such miRs that regulate the neuroimmunological processes could lead to exploring their potential as therapeutics (Soreq and Wolf, 2011).

### miRNA and circadian rhythms in hypoxia/ischemia

Superchiasmatic nucleus (SCN) located in the anterior hypothalamus of a mammalian brain is the central switch for circadian rhythms. Apart from SCN, it has been demonstrated that each cell in the body has its own clock which controls the molecular and cellular functions. The proteins associated with the molecular clock include, Brain, Muscle ARNT-Like protein 1 (Bmal1), Period (Per1, Per2), Cryptochrome (Cry), and Circadian Locomotor Output Cycles Kaput (CLOCK) (Shearman et al., 1997; Zylka et al., 1998). All these proteins belong to the bHLH-PAS protein family which is characterized by the presence of basic helix-loop-helix domain. The members of this protein family are known to play an important role in response to low oxygen as well as diurnal changes in light. Another protein that belong to this same family includes HIF1α that is involved in the maintenance of oxygen homeostasis. Another new proteins have been recently identified, MOP3 and MOP9, which are associated with both the pathways, i.e., circadian and hypoxia (Bunger et al., 2000; Hogenesch et al., 2000).

The variables that display circadian patterns include body temperature, metabolic rate etc. Any condition that causes prolonged hypoxia ultimately disturbs the oscillations of these essential parameters and their associated body functions. Under normal physiological conditions, these events follow well-defined oscillations which help in maintenance of homeostasis. Studies have demonstrated the existence of a cross-talk between these two pathways (Ghorbel et al., 2003). Chilov et al. in their study observed increase in the level of PER1 and CLOCK along with co-immunoprecipitation of HIF1α with PER1 under hypoxia in mice (Chilov et al., 2001).

Circadian rhythms have also been associated with stroke occurrence in humans with the temporal and seasonal pattern of occurrence (Marler et al., 1989; Argentino et al., 1990; Elliott, 1998; Raj et al., 2015). Higher risk for hemorrhagic stroke has been reported in evening (Elliott, 1998). In another study, higher frequency of ischemic stroke was noted in 6 a.m. to noon quarter as compared to other quarters of the day (Argentino et al., 1990). These studies show that the circadian variation in time of occurrence can depend on the sub-type, which can also help in planning therapeutics.

miRNA regulation of circadian rhythms has also been implicated through different studies (Chen and Rosbash, 2016; Gao et al., 2016). It binds to 3′ UTR region of the target genes and control different processes such as its mRNA stability and/or translation. The miRNA that control the circadian rhythms, such as miR219, miR132, also show rhythmic expression in SCN. Cheng et al. in their study have demonstrated increase in rhythmicity as an outcome of administration of antagonists against these miRs (Cheng et al., 2007). In another study by Shende et al. investigated the miRNAs that target the clock gene, Bmal1, in mouse which were exposed to 12 h LD cycles. The authors identified miR 152, miR 494, which showed bimodal pattern of expression, following diurnal oscillations along with the levels of Bmal1. These miRs were also shown to interact with Clock (Shende et al., 2011).

Many physiological features, especially cardiovascular, follow defined circadian cycles. Therefore, the circulating levels of miRNA play an important role in circadian control associated with different pathology. The different identified miRNAs can also act as targets as well as biomarkers for any disturbances from normal physiology. The knowledge about the miRNA involved in the clock genes is still limited and needs further detailed investigations for their translation to clinics.

## Overcoming hypoxia with yoga based alternate therapies

With increasing evidence in the past years, Yoga is gaining recognition as a therapeutic intervention in the management of non-communicable diseases like hypertension, coronary artery disease (Cramer et al., 2014), diabetes (Innes and Vincent, 2007), obesity, back pain (Cramer et al., 2013), and also as an adjunct in the management of cancer. The reduction in respiratory rate, heart rate, vagal predominance as a general response following yoga practices possess a considerable role in managing hypoxia and its associated pathophysiological responses. This understanding might be beneficial in its application as a targeted therapy. While meditation's any effect on Alzheimer disease (AD) is hypothetical, meditation has received significant consideration as a tool that possibly will have positive medical and psychological benefits. Studies indicate improvement in cognitive functions in elders with dementia (Oken et al., 2006). Kirtan Kriya (KK), a type of meditation was also investigated in subjects with memory impairment, Newberg et al. demonstrated positive effects of KK on cerebral blood flow and cognitive function. A difference in the activation of the anterior cingulate gyrus and prefrontal cortex, was seen in the subjects who practiced KK (Newberg et al., 2010). Neuroimaging studies following Yoga practices in individuals with mild cognitive impairment suggest increased memory performance. Enhanced memory performance was correlated with increased neuronal connectivity between default mode networks and frontal medial cortex, pregenual anterior cingulate cortex, right middle frontal cortex, posterior cingulate cortex, and left lateral occipital cortex suggesting that yoga might be helpful in enhancing memory recall, specifically visual memory encoding (Eyre et al., 2016). Meditation improves sleep and sleep problem is also a risk factor for AD (Devore et al., 2014; Innes and Selfe, 2014). Froeliger et al. revealed that upon conducting controlled breathing exercises such as pranayama and meditation, there was a significant decrease in cognitive failures as evaluated by Voxel-Based Morphometric analysis, which showed a positive co-relation with increased GMV (Gray matter volume) in the regions of cerebellar, temporal, occipital, limbic, and frontal lobe of the brain. There was also a positive correlation seen with the amount of increase in GMV and the duration of practice of the breathing exercises and meditation (Froeliger et al., 2012). Long term meditation practice is associated with larger overall gray matter volume and increased regional enlargement of areas associated with sustained attention, introspection, and autonomic function surveillance, indicating the possible role of Yoga practices in facilitating neuroplasticity (Hernández et al., 2016). It has also been seen in studies that upon activities that increase cerebral perfusion as well as increase oxygen inhalation, such as performing yogic asanas (yogic poses) and pranayama (yogic breathing exercises), there is an increase in the amplitude in the P3 wave evoked potential (Tripathi and Bharadwaj, 2013). This escalation in P3 amplitude means that there is an increased neuronal pool recruitment, which can be seen as increased attention as well as memory updating. The study revealed that those who took part in activities which, increased cerebral perfusion showed a significantly improved functioning in comparison to the control group which was only on oral Alzheimer medications. Sutton et al. first of all identified the P3 wave in 1965 and since then it has been used extensively in the area of event-related potentials (ERPs) (Sutton et al., 1965). ERPs are electrophysiological responses produced in the brain as a result of specific events or stimuli (Blackwood and Muir, 1990). The latency range for most adults for auditory stimuli is 250–400 ms. The latency is generally explained on the basis of the speed of stimulus of one event from another. Shorter latencies signify exceptional mental execution compared to longer ones. The P300 can be employed as a diagnostic tool to discriminate dementias of different origin and can be elicited by a wide variety of sensory or motor events. In addition, P300 is used to evaluate and monitor Alzheimer's disease (AD). Lengthening of latency is the most pronounced variation in the auditory modality in AD and other cortical dementias, which is related to debilitated memory (Polich et al., 1990). Latency lengthening is also observed in AD with visual modality stimulation and in the auditory modality in healthy individuals with increased risk of having AD. This may be used in early monitoring for this disease (Saito et al., 2001). In related investigation by Caravaglios et al. it was revealed that P300 latency was higher in AD elderly subjects as compared to controls (Caravaglios et al., 2008). A similar study found significant differences in P300 latency between AD subjects and control (O'mahony et al., 1996). Lai et al. determined P300 latency as well as amplitude in AD group, group with mild cognitive impairment, and the control group (Lai et al., 2010). The authors found that P300 latency was higher in AD group followed by those with cognitive involvement as compared to controls, while no difference in P300 amplitude was observed among the three groups. Similarly in another study Yamaguchi et al. compared P300 latency and amplitude in Alzheimers' with vascular dementia and the controls and found that both the diseased groups had a higher latency as compared to the control group (Yamaguchi et al., 2000). Several other studies evaluated P300 latency and its subcomponents, called P3a and P3b in AD patients and compared them with controls and found significantly higher latency in patients compared to controls (Frodl et al., 2002; Bennys et al., 2007; Juckel et al., 2008). Telles et al. assessed whether practicing alternate nostril yoga breathing (*nadisuddhi pranayama*) has any influence on P300 auditory evoked potentials and found a dramatic elevation in the P300 peak amplitudes suggesting that it unequivocally affects intellectual processes (Telles et al., 2013; Mccaffrey et al., 2014), conducted a study and found the possibility of older adults with AD to complete the Sit “N” Fit Chair Yoga Program with positive changes across each physical process (Mccaffrey et al., 2014). In healthy individuals, controlled breathing at a rate of 5.5 breaths per minute evoked activity at the brainstem, across the dorsal length of pons, in hypothalamus, hippocampus, lateral cortices and regions of striatum. Interestingly, studies on meditation techniques (Cyclic Meditation and Transcendental Meditation) in healthy regular practitioners demonstrated hypometabolic states with significant reductions in oxygen consumption (Wallace, 1970; Telles et al., 2013). Long-term Yoga practices are independently associated with decreased chemoreflex hypoxic and hypercapnic responses and better baroreflex sensitivity (Spicuzza et al., 2000). This might be achieved as a result of adaptation of peripheral and central chemoreceptors and pulmonary stretch receptors to the regular practice of slow breathing during yoga practices, followed by a decrease in the vagal afferent discharge to the bulbopontine centers. Hypometabolic state in yoga practitioners could enable tolerating hypoxic conditions. Another study designed to understand the efficacy of Yoga practices in promoting physiological recovery showed that Yoga practitioners with 2 years of experience practicing yoga atleast twice a week produced 41% less lipopolysaccharide stimulated IL-6 in response to the laboratory stressor than novices (Kiecolt-Glaser et al., 2010). Investigations are required to understand the regulatory role of Yoga on miRNA. However, based on the literature available, we speculate that miRNA associated in the process of inflammation, angiogenesis and stress response might be differentially regulated in long term practitioners, whereas, the same is expected be the process through which the therapeutic effects are moderated by Yoga. Therefore, Yoga practices might be a promising tool to understand the inherent ability of the body in managing hypoxia induced CNS injuries and also as a possible therapeutic tool.

## Future directions

With advancement in awareness about the profiling and expression patterns of different miRNA associated with hypoxia/ischemia-induced CNS injuries, it has led to translation of this information in clinical set-ups. The miRNA levels could now be utilized as clinical biomarkers for ischemia. Moreover, since a single miRNA could regulate a whole network with multiple targets, controlling the levels of individual miRNA species could assist in designing therapeutics for complex diseases. Unlike other exercises, Yoga practices appear to be unique and the present day findings indicate that yoga can restore or maintain homeostasis. However, the underlying mechanisms need to be understood in detail. Further investigations pertaining to miRNAs linked with ischemic injuries and the role of Yoga practices in regulating them could benefit in management and therapy for CNS pathologies in future.

## Author contributions

GM wrote the manuscript and contributed in writing content related to hypoxia in CNS pathologies, miRNA in neuroprotection, and circadian rhythms in hypoxia etc.; DM contributed in writing content related to hypoxia pathophysiological responses and the role of miRNA in inflammation, angiogenesis, and neuroprotection; BR contributed content for the Yoga based therapeutics; VP added yoga related information in the manuscript; NS added information related to correlation between cerebrovasculature and AD pathogenesis, yoga related studies, and hypoxia in promoting cell differentiation; AA conceptualized, designed, and edited the manuscript.

### Conflict of interest statement

The authors declare that the research was conducted in the absence of any commercial or financial relationships that could be construed as a potential conflict of interest.
